# COVID-19 and vaccination induced changes in hospital activity in Malta, Q1 2020 to Q1 2021: a population-based study

**DOI:** 10.1186/s42506-021-00101-1

**Published:** 2022-02-08

**Authors:** Sarah Cuschieri, David Borg, Steve Agius, Hagen Scherb, Victor Grech

**Affiliations:** 1 Faculty of Medicine and Surgery, University of Malta, RM425 Biomedical Building, Msida, MSD2080 Malta; 2 Clinical Performance Unit, Mater Dei Hospital, Msida, Malta; 3 Administration, Mater Dei Hospital, Msida, Malta; 4 German Research Center for Environmental Health, Institute of Computational Biology, Ingolstädter Landstr. 1, D-85764 Neuherberg, Germany; 5 Department of Paediatrics, Mater Dei Hospital, Msida, Malta

**Keywords:** Coronavirus infections, Hospital admissions, Pandemic, Morbidity, Mortality, Mass vaccination, Malta

## Abstract

**Background:**

COVID-19 has severely impacted global healthcare services. Malta has only one acute state hospital, Mater Dei Hospital (MDH), and at the time of writing is the most vaccinated country in Europe. Malta thus provides an ideal setting to assess the impact of COVID-19 on healthcare services at population level, including the impact of vaccination on hospital admissions.

**Methods:**

Hospital data was obtained as anonymised totals from MDH’s Clinical Performance Unit and the European Centre for Disease Prevention and Control. COVID-19-related data was obtained from the Ministry of Health dashboard. Comparative assessments were performed to explore associations between the COVID-19 situation, vaccination, and hospital activity. Poisson regression was used to model the counts of monthly accident and emergency (A&E), outpatient clinics attendances and hospital admissions.

**Results:**

A&E, hospital admissions, and outpatient clinics attendances declined (31.88%; 23.89%; 29.57%; *p* < 0.01 respectively) with onset of COVID-19 till April 2021 when compared to pre-COVID years (2017–2019). Admissions due to COVID-19 initially increased in parallel to the population’s COVID positivity. Vaccination rollout led to a decline in COVID-19 admissions.

**Conclusions:**

The drastic drop in admissions and outpatient attendees was expected but not for A&E attendees as acutely ill patients should still have attended. This is of public health concern since delayed or deferred medical management increases population morbidity, mortality and increases the eventual burden on the healthcare system. Mass vaccination saw the return to normality with an increase in A&E burden.

**Supplementary Information:**

The online version contains supplementary material available at 10.1186/s42506-021-00101-1.

## Introduction

COVID-19 has ravaged the world for over a year and infected over 560,000,000 individuals at the time of writing (20th June 2021) [1]. Healthcare services have been severely impacted, with some countries actually collapsing and some others reporting decline in hospital activities [2–4].

Malta is one of the smallest countries in the world and is comprised of a small archipelago in the centre of the Mediterranean with a total population of almost 500,000, a land area of 316 km^2^ and has the 7th highest population density in the world. Only one large National Health Service Hospital with intensive care facilities (Mater Dei Hospital) services the nation along with a few other much smaller primary care facilities and private hospitals [5]. The first COVID-19 case was identified in Malta on the 7th of March 2020 [6, 7]. The first wave of infection was well contained unlike the second wave [6, 8]. Hospital preparations for an inevitable pandemic outbreak started months before the actual onset of COVID-19 in Malta, which prevented the healthcare system from being overwhelmed along with implementation of various mitigation measures [9]. Later on, when COVID-19 vaccination was approved for rollout, a vaccination logistics team was set up along with infrastructural changes to accommodate mass population vaccination in Malta [10].

The aim of this study was to assess the impact of COVID-19 over a period of a year, on the healthcare services (accident and emergency, hospital admissions and outpatient clinics) at a population level, while comparing to the local COVID-19 situation. Furthermore, this study explored the impact of vaccination on hospital admissions. The outcome of this population-based study is vital for both the local and international public health and hospital administration bodies. Malta is a known cardiometabolic country and is currently (up till time of writing) the highest COVID-19 vaccinated country in Europe; hence, this study’s outcomes act as a good proxy for other countries, large and small, for the effect of vaccination on hospital activity [11–15].

## Methods

### Study design

A retrospective observational study was conducted on anonymised data originating from the only state acute hospital of Malta, Mater Dei Hospital.

### Data

Mater Dei’s Clinical Performance Unit provided anonymised totals of the national hospital data for the Accident and Emergency (A&E) attendances, outpatient attendances and hospital admissions, including in-patient overnight (elective and emergency) admissions and day cases. Detailed data was available from the year 2017 up to April 2021. Monthly COVID-19-positive cases, intensive critical unit admissions, and vaccination doses were obtained from Malta’s Ministry of Health COVID-19 dashboard, local newspaper articles and One World in Data (OWID) [7, 16, 17]. Monthly data on COVID-19 admissions to hospital wards was obtained from the European Centre for Disease Prevention and Control (ECDC) [18]. Annual population statistics were obtained from official annual publications of the National Statistics Office of Malta [19].

### Comparative assessment

Monthly hospital admissions from January 2020 till April 2021, total COVID-19-positive cases, COVID-19 admissions, and cumulative vaccination doses were converted to represent the population per 100,000. Graphical comparative assessments were performed to explore for trends between these variables across time (January 2020 up to April 2021).

### Statistical analyses

The counts of hospital admissions and outpatient attendances were considered Poisson distributed random variables. Consequently, Poisson regression with effect coding was used to model the temporal trends of the absolute counts of admissions and attendances. Regression models were adjusted for categorial dummy variables representing seasonality (months) and lockdown periods. Basic descriptions and regression analyses were stratified by and adjusted for medical or clinical specialties. The continuous variable ‘people fully vaccinated per hundred’ of the publicly available OWID data set was employed as an additional potential determinant of the trends. A delay of 2 weeks after the second vaccination for presumably somewhat stronger vaccination effects or side-effects was considered by way of trial. The interactions of all 12 months with time were included for a full seasonality adjustment. If in the Poisson regression analyses the ‘deviance goodness-of-fit statistics (=deviance/degrees of freedom)’ was significantly greater than 1, correction for over-dispersion was employed: SAS procedures’ option ‘scale=d’. To be conservative in this respect, no correction was done in the (rare) cases of under-dispersion. Software used was MS-Excel-365 (2016), Wolfram MATHEMATICA 11.3, and mostly procedures GENMOD and SGPLOT of SAS/STAT software 9.4, SAS Institute Inc.: SAS/STAT User’s Guide, Cary NC: SAS Institute Inc., 2014.

## Results

Since the onset of COVID-19 in Malta, two soft lockdowns were instituted in March 2020 and March 2021, with a total of 30,292 individuals reported to have tested positive by the end of April 2021 [6, 20]. As part of the first lockdown (March 2020) mitigation measures, all non-essential healthcare services were suspended, with postponement or cancellations of outpatient consultations and elective surgeries [6]. These were slowly restarted during the transition period (May 2020) while following mitigation measures [21]. On the 10th of March 2021, the second lockdown was announced, but only elective surgeries were postponed, leaving outpatients operational [20].

### Accident and emergency (A&E) attendances

**Fig. 1:**
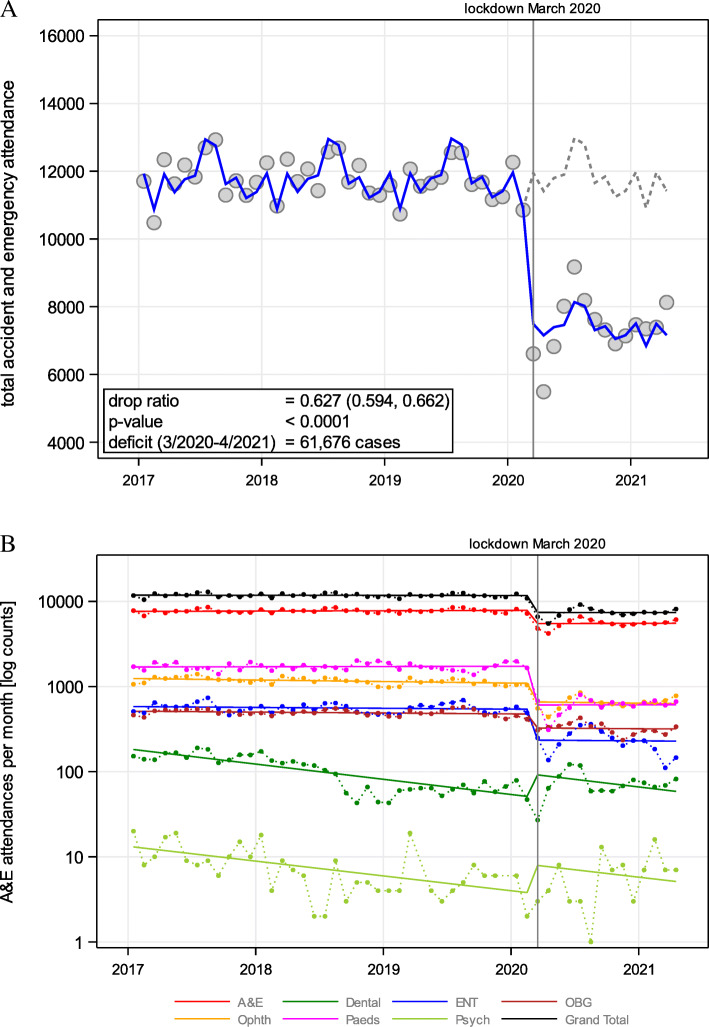
Time trend of (**A**) total A&E attendance from 2017 to April 2021; and (**B**) according to specialty and Poisson regression from March 2020 onwards

During the pre-COVID-19 years of 2017, 2018, and 2019, A&E attendances were 141,758, 142,519, and 140,209, respectively. Supplement Table 1 provides a breakdown of the attendees between 2017 and April 2021. For the year 2020 (COVID-19 era), the total A&E attendance experienced a significant drop (*p* < 0.01) of 31.88% (total attendance *n* = 96,382) when compared to the average A&E attendances for 2017–2019, as shown in Fig. 1A. The highest drop in attendance was observed for the months of March and April, after which the A&E attendances climbed in a fluctuant trend. However, the overall A&E attendance between March 2020 and April 2021 did not reach the pre-COVID-19 numbers.

On stratifying the A&E attendances by the different A&E sub-speciality services, a similar trend could be observed (Fig. 1B). A significant drop in attendances was established for ear-nose, and throat (ENT) A&E, obstetric (OBS) A&E, ophthalmic (Opthth) A&E, and paediatrics (Paed) A&E (*p* < 0.01 respectively; Supplement Table 2). However, no (significant) attendance drops were established for psychiatric and dental services (Supplement Table 2). Indeed, positive increases were observed of + 115.4% (CI 95%: − 58.2 to + 1009.1) for psychiatry and + 85% (CI 95%: + 17.3 to + 191.8) for dental services.

### Hospital admissions

**Fig. 2:**
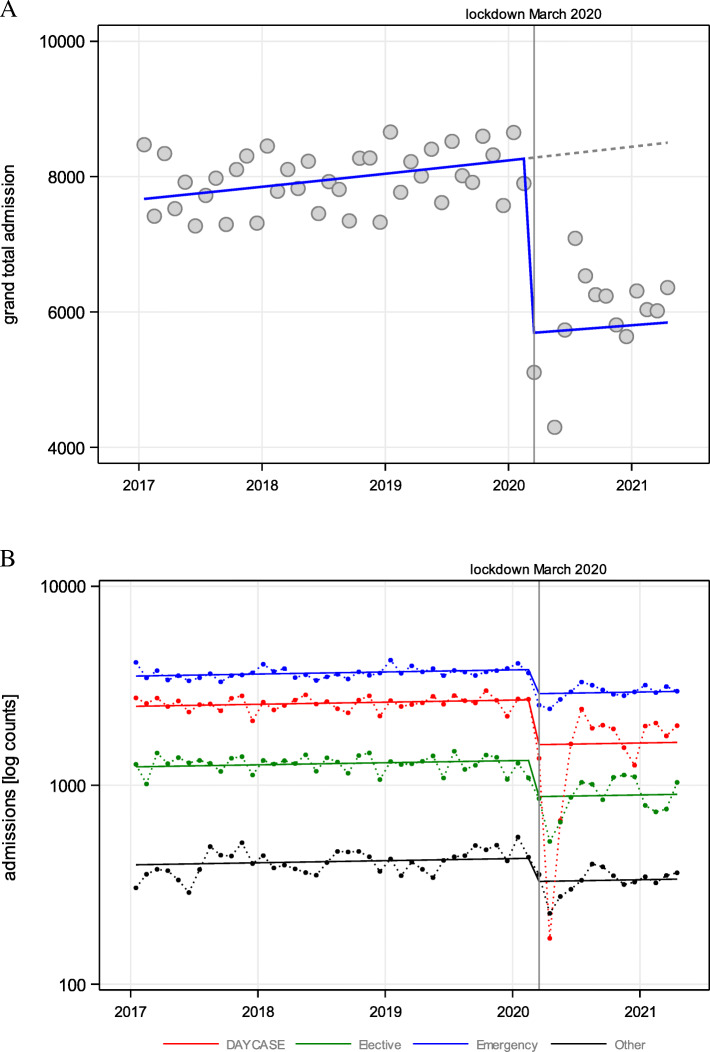
Time trend of (**A**) total hospital admissions from 2017 to April 2021; and (**B**) according to different categories and Poisson regression from March 2020 onwards

Hospital admissions for 2017, 2018, and 2019 were 93,658; 94,803; and 97,618, respectively, with admissions dropping from March 2020. Supplement Table 3 provides a breakdown of the admissions between 2017 and April 2021, and analysis of maximum likelihood parameter estimates. Indeed, total admissions dropped by 23.89% in 2020 (total admissions *n* = 72,581), with a significant drop following the March 2020 lockdown (*p* < 0.01). April 2020 recorded the lowest number of admissions, followed by a gradual increase across the subsequent months. However, admissions did not reach the pre-COVID-19 numbers at any point across 2020 to 2021 (Fig. 2A). A similar drop in admission trends (2017 to 2020) could be observed on stratifying the admissions by different categories; day case, elective, emergency, and other (*p* < 0.01), as shown in Fig. 2B.

**Fig. 3:**
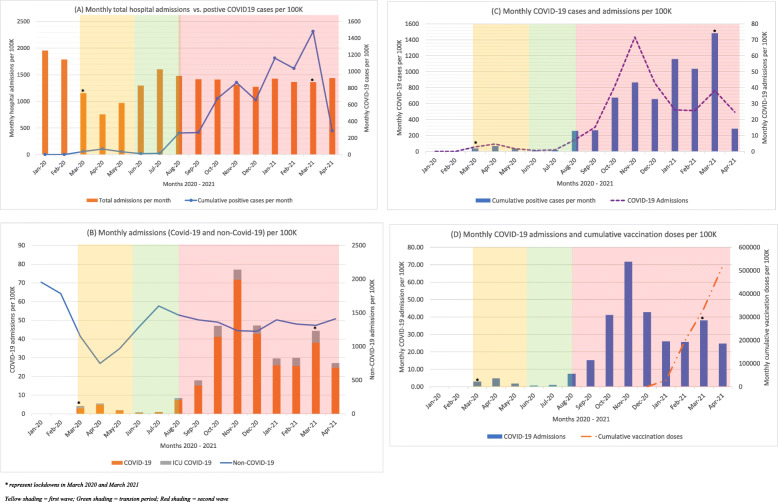
Comparisons between: (**A**) montly total hospital admissions and COVID-19 cumulative cases; (**B**) montly admissions stratified according to COVID-19 or not; (**C**) montly cumulative positive COVID-19 cases and COVID-19 admissions; and (**D**) monthly COVID-19 admissions and cumulative COVID-19 vaccination doses

As population cumulative COVID-19-positive cases rose across the second wave, total hospital admissions remained relatively stable (Fig. 3A). On stratifying the admissions according to having COVID-19 or not, it was observed that admissions due to COVID-19 were predominant across the second wave, reaching a peak in November 2020, as shown in Fig. 3B. Indeed, the admissions due to COVID-19 increased simultaneously as the population’s positive COVID-19 cases increased (Fig. 3C). However, as of December 2020, this positively correlated relationship changed to a negatively correlated one, where even though population cumulative positive COVID-19 cases increased, the hospital admissions due to COVID-19 started to decline (Fig. 3C). This corresponds to the initiation of the COVID-19 vaccination programme in Malta, as shown in Fig. 3D.

### Outpatient clinics attendances

**Fig. 4:**
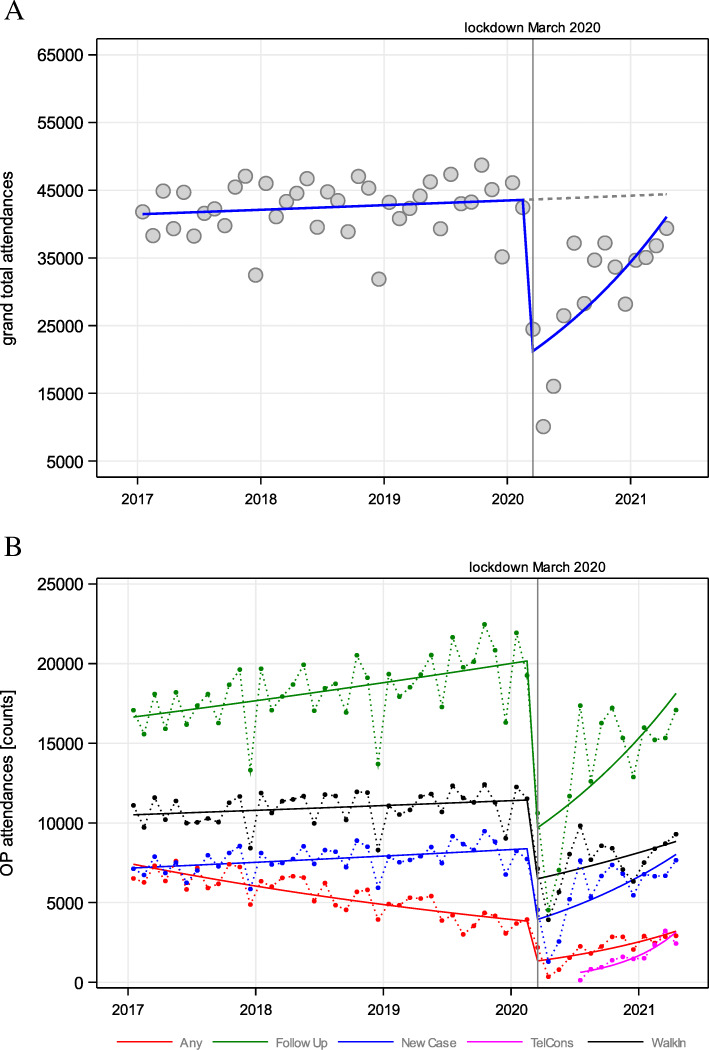
Time trend of (**A**) total hospital outpatient attendees from 2017 to April 2021; and (**B**) according to different categories and Poisson regression from March 2020 onwards

Total outpatient clinics attendances have seen a gradual increase across the pre-COVID-19 years with a total of 495,816 attendances in 2017, 512,535 in 2018 and 518,589 in 2019. However, when comparing to 2020, a significant (29.57%) drop in physical outpatient clinic attendance was noted (*p* < 0.01; *n* = 358,453), as shown in Fig. 4A. A similar significant trend was present on stratification by the different outpatient cases (new, follow-up, walk-in, telemedicine; *p* < 0.01 respectively) as shown in Fig. 4B. Supplement Table 4 provides a breakdown of the outpatient attendances between 2017 and April 2021 and the analysis of maximum likelihood parameter estimates.

### Vaccination and A&E activity

**Fig. 5:**
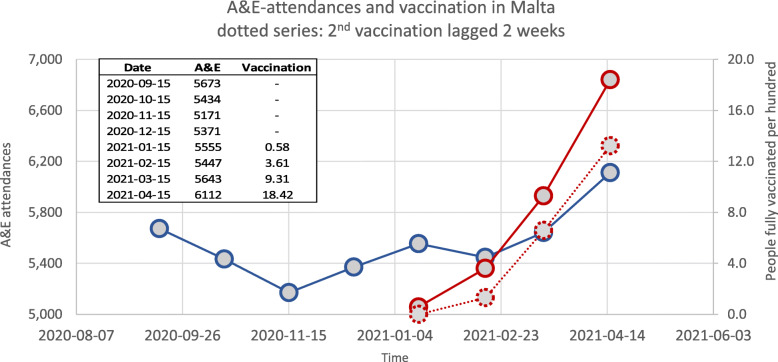
A comparison analysis between A&E attendees and the cumulative vaccination doses per 100 population

Vaccination rollout in Malta was initiated at the end of December 2020, like the rest of the European Union countries [22]. Figure 5 shows a comparison analysis between A&E attendees and the cumulative vaccination doses per 100 population. A significant relationship was observed between the cumulative vaccination and the overall A&E admissions (*p* = 0.02) as well as when compared to the monthly adjusted trend model for A&E, as was shown in Fig. 1A (*p* = 0.0003). Indeed, with every 1% vaccination increase, the absolute numbers of A&E attendees increased by 0.9%. On assessing for a delay of 2 weeks after the second vaccination for presumably somewhat stronger vaccination effects or side-effects, a stronger statistically significant relationship was established (*p* = < 0.0001). Supplement Figure 1 and 2 shows the A&E attendance by vaccination dynamic and Poisson regression trends, with and without the 2-week lag. No significant relationship was present when stratifying A&E attendees by specialities with cumulative vaccination (*p* = 0.41).

## Discussion

COVID-19 positivity is typically followed by a rise in admissions after several days and deaths a few days thereafter [23, 24]. Similar to other countries, non-essential healthcare services were postponed or cancelled a few days after onset of COVID-19 in Malta [6, 25]. The principal aim was to avoid overwhelming the only state hospital, while shifting human resources to treat COVID-19 patients [6]. This is clearly reflected in this study’s observations in the drastic drop in admissions and outpatient attendees in March and April of 2020.

Although A&E services remained operational as before the onset of COVID-19, a decrease in attendees was also noted, coinciding with other studies [3, 4, 26, 27]. This can be attributed to fear of contracting SARS-CoV2 infection as well as reflecting balancing act in the medical professional decision-making between the risk of contracting SARS-CoV2 infection and risk of health deterioration of their patients [3]. Other potential reasons could be the enactment of the Maltese “Protection of Vulnerable Persons Order, Public Health Act” during the first wave, where vulnearble people were coocooned with less chance of contracting not only SARS-CoV2 infection but also other infectious diseases [28, 29]. The same principle is applicable for children as closure of schools broke potential viral school transmission [30, 31]. Indeed, this is reflected in the low positive COVID-19 cases over the first wave in Malta [6]. Additionally, patients may have found alternate ways to seek medical advice or treatment without visiting the hospital through their general practitioner (GP) or telemedicine. In fact, Mater Dei Hospital initiated a new service of telemedicine from July 2020, as a means of decreasing outpatient attendees, as seen in this study. However, this might have resulted in some acute patients being missed which would have otherwise been identified in a face-to-face consultation and either referred to A&E or had a direct admission from the outpatient clinic [32].

The global decrease in A&E attendances when compared to the pre-COVID-19 years, even when the healthcare services were restored to their normal activity and the positive case numbers stabilised [33], presents a paradox [34]. Acutely ill patients should still have attended A&E regardless of the COVID-19 situation. This is especially since Malta has an ageing population, with 33% of the adult population suffering from multimorbidity (two or more concurrent chronic diseases) [14, 19]. This sharp decline is of public health concern since delayed or deferred medical management of serious conditions increases the morbidity and mortality while increasing further the burden on the healthcare system when these patients are eventually admitted to the hospitals. Deferring medical attention can be expected to lead to an increase in the proportion of missed medical diagnoses and the new onset of chronic diseases. Indeed, this has already been reported in the UK with reductions in monitoring and diagnosis of type 2 diabetes and a potential high prevalence of unknown new onset diabetes [35]. A similar situation may be present for Malta, especially since in the year 2016, it was already reported that 4% of the adult population were unaware of their type 2 diabetes [13]. It is anticipated that a surge in attendees will be observed, most likely with a late diagnosis or complications, in the unforeseen future.

Of note, our study observed an increase in attendees for the psychiatric A&E service as opposed to other speciality services, contrary to findings in other countries [36, 37]. However, our results are in accordance with the indirect psychological impact that COVID-19 has inflicted on the general population, COVID-19 cases, and individuals with known mental health illness [38–41]. Indeed, it has been reported that COVID-19 has increased concerns for livelihoods, employment redundancy, and elevated levels of domestic violence especially related to increased alcohol consumption during lockdowns [42, 43].

### Effect of vaccination on hospital activity

The vaccination programme in Malta prioritised the elderly (80+ years) and healthcare workers then progressively invited adults in descending age order [44]. As the rapid vaccination rollout progressed, this study showed a progressive decline in COVID-19 admissions. In fact, it has been reported that following just one dose of Pfizer (BNT162b2) or AstraZeneca (ChAdOx1-S) vaccines, the risk of emergency admissions due to COVID-19 was reduced by 43% and 37% respectively, in a UK study [45]. Similar results were noted in the USA [46].

A&E attendees showed a slight increase as the vaccination rollout progressed. During this time frame, the population began to gradually return to normality with a shift from remote working and schooling to physical presence, reopening of non-essential shops and a general sense of security. This increased the risk of workplace and road traffic accidents back to pre-COVID levels and led to the normal return of transmission of other infectious diseases. However, one cannot exclude the possibility of COVID-19 vaccination side effects or the concern of such side effects having contributed to a proportion of this rise in A&E burden.

### Implications for practice and policy

The COVID-19 pandemic has challenged all healthcare systems and has brought to light the importance of hospital preparedness for outbreaks. The shifts in hospital activity should be considered and implemented in new forecasting models for future planning. However, it is essential for policy makers (public health and hospital) to target both the pandemic, in this case COVID-19 and other pre-existing epidemics, such as type 2 diabetes, cardiovascular disease, and others. Diverting hospital care to just the outbreak will result in a surge in chronic diseases and epidemic later. It is therefore recommended that strategies are implemented that engage communities’ outreach and educational campaigns to ensure that all patients receive medical care in a timely manner. Homecare health initiatives may also be another way to target and care for vulnerable populations [3]. Even though there may be some rare vaccine side effects occurrences, it is still recommended to advocate for mass population vaccination since overall, this has decreased morbidity and mortality, and enabled the gradual return to normality

However, vaccination should not replace the mitigation measures, such as physical distancing, as complete relaxation at this point in time can lead to resurgences of infections, emergence of new variants, and an increase in hospitalisations especially among the comorbid, vulnerable and elderly [47]. Another aspect to be considered is the substantial impact the pandemic has had on society, psychological health, and economies among others. The pandemic’s impact on mental health is evident in this study, which if neglected might have a long-term morbidity impact on the population [48]. The early detection of psychological crises or problems among the general population through online surveys and in healthcare clinics, the engagement of population based psychoeducation and increased access to healthcare professionals are some of the proposed actions to target this emerging population burden [49].

### Study strengths and limitations

This is a population-based study which analysed the only state hospital in Malta, hence provides an insight of the impact of COVID-19 at a population level. Seasonality effects were considered in the regression analyses to provide more on-point statistical comparisons. However, this is a retrospective observational study with no data on the clinical diagnoses and reasons for A&E attendance and admissions. Hence, the study was unable to identify and correlate the reason/diagnosis with the hospital services’ trends and had to rely on assumptions.

## Conclusions

The drastic drop in admissions and outpatient attendees was expected but not for A&E attendees as acutely ill patients should still have attended. This is of public health concern since delayed or deferred medical management increases population morbidity and mortality and increases the eventual burden on the healthcare system. Mass vaccination saw the return to normality with an increase in A&E admissions.

## Supplementary Information

**Additional file 1: Supplement Table 1.** Breakdown of the Accident and Emergency attendees at Mater Dei Hospital between 2017 and April 2021. **Supplement Table 2.** (A) Breakdown of the Accident and Emergency attendees at Mater Dei Hospital between 2017 and April 2021 by specialities (B) Analysis of maximum likelihood parameter estimates for level shifts. **Supplement Table 3.** (A) Breakdown of the hospital admission to Mater Dei Hospital between 2017 and April 2021 by different categories (B) Analysis of maximum likelihood parameter estimates. **Supplement Table 4.** (A) Breakdown of the outpatient clinic attendees to Mater Dei Hospital between 2017 and April 2021 by different categories (B) Analysis of maximum likelihood parameter estimates. **Supplement Figure 1.** (A) A&E attendance by vaccination dynamics and (B) Analysis of maximum likelihood parameter estimates. **Supplement Figure 2.** (A) A&E attendance by vaccination dynamics with a two week lag and (B) Analysis of maximum likelihood parameter estimates

## Data Availability

Data is available upon request.

## Abbreviations

A&E: Accident and Emergency department
COVID-19: Coronavirus disease 2019
ENT: Ear-nose and throat
GP: General practitioner
Paed: Paediatrics
OBS: Obstetric
Opthth: Ophthalmic
OWID: One world in data
UK: United Kingdom
