# Supplementary material for: COVID-19 and vaccination induced changes in hospital activity in Malta, Q1 2020 to Q1 2021: a population-based study
Source: J Egypt Public Health Assoc. 2022 Feb 8;97:7. doi: 10.1186/s42506-021-00101-1 (PMC8825920; doi:10.1186/s42506-021-00101-1)
Supplement: Supplementary file 1 — Additional file 1: Supplement Table 1. Breakdown of the Accident and Emergency attendees at Mater Dei Hospital between 2017 and April 2021. Supplement Table 2. (A) Breakdown of the Accident and Emergency attendees at Mater Dei Hospital between 2017 and April 2021 by specialities (B) Analysis of maximum likelihood parameter estimates for level shifts. Supplement Table 3. (A) Breakdown of the hospital admission to Mater Dei Hospital between 2017 and April 2021 by different categories (B) Analysis of maximum likelihood parameter estimates. Supplement Table 4. (A) Breakdown of the outpatient clinic attendees to Mater Dei Hospital between 2017 and April 2021 by different categories (B) Analysis of maximum likelihood parameter estimates. Supplement Figure 1. (A) A&E attendance by vaccination dynamics and (B) Analysis of maximum likelihood parameter estimates. Supplement Figure 2. (A) A&E attendance by vaccination dynamics with a two week lag and (B) Analysis of maximum likelihood parameter estimates [file 42506_2021_101_MOESM1_ESM.docx]

Supplement Table 1. Breakdown of the Accident and Emergency attendees at Mater Dei Hospital between 2017 and April 2021

|  | **2017** | **2018** | **2019** | **2020** | **2021** |
| --- | --- | --- | --- | --- | --- |
| **January** | **11706** | **12248** | **11591** | **12264** | **7466** |
| Other | 382 | 312 | 449 | 327 | 244 |
| Doctor | 2875 | 2895 | 2781 | 2622 | 1371 |
| Self-referral | 8449 | 9041 | 8361 | 9315 | 5851 |
| **February** | **10480** | **10976** | **10737** | **10853** | **7346** |
| Other | 360 | 340 | 439 | 278 | 254 |
| Doctor | 2468 | 2576 | 2475 | 2454 | 1201 |
| Self-referral | 7652 | 8060 | 7823 | 8121 | 5891 |
| **March** | **12342** | **12356** | **12063** | **6609** | **7391** |
| Other | 420 | 320 | 412 | 365 | 325 |
| Doctor | 2815 | 2784 | 2624 | 1408 | 1215 |
| Self-referral | 9107 | 9252 | 9027 | 4836 | 5851 |
| **April** | **11626** | **11692** | **11554** | **5489** | **8126** |
| Other | 365 | 322 | 445 | 561 | 311 |
| Doctor | 2574 | 2656 | 2795 | 651 | 1501 |
| Self-referral | 8687 | 8714 | 8314 | 4277 | 6314 |
| **May** | **12182** | **12066** | **11650** | **6823** |  |
| Other | 436 | 311 | 454 | 495 |  |
| Doctor | 2547 | 2795 | 2606 | 771 |  |
| Self-referral | 9199 | 8960 | 8590 | 5557 |  |
| **June** | **11831** | **11426** | **11821** | **8013** |  |
| Other | 350 | 372 | 446 | 451 |  |
| Doctor | 2487 | 2426 | 2523 | 962 |  |
| Self-referral | 8994 | 8628 | 8852 | 6600 |  |
| **July** | **12695** | **12570** | **12551** | **9172** |  |
| Other | 370 | 298 | 402 | 336 |  |
| Doctor | 2507 | 2593 | 2698 | 1247 |  |
| Self-referral | 9818 | 9679 | 9451 | 7589 |  |
| **August** | **12925** | **12681** | **12543** | **8184** |  |
| Other | 372 | 382 | 440 | 252 |  |
| Doctor | 2513 | 2597 | 2592 | 1183 |  |
| Self-referral | 10040 | 9702 | 9511 | 6749 |  |
| **September** | **11296** | **11683** | **11610** | **7620** |  |
| Other | 344 | 329 | 352 | 249 |  |
| Doctor | 2308 | 2392 | 2635 | 1292 |  |
| Self-referral | 8644 | 8962 | 8623 | 6079 |  |
| **October** | **11717** | **12172** | **11679** | **7313** |  |
| Other | 386 | 300 | 365 | 315 |  |
| Doctor | 2521 | 2740 | 2609 | 1194 |  |
| Self-referral | 8810 | 9132 | 8705 | 5804 |  |
| **November** | **11287** | **11356** | **11162** | **6905** |  |
| Other | 378 | 446 | 360 | 211 |  |
| Doctor | 2419 | 2549 | 2379 | 1179 |  |
| Self-referral | 8490 | 8361 | 8423 | 5515 |  |
| **December** | **11671** | **11293** | **11248** | **7137** |  |
| Other | 381 | 464 | 382 | 232 |  |
| Doctor | 2345 | 2379 | 2307 | 1176 |  |
| Self-referral | 8945 | 8450 | 8559 | 5729 |  |
| **Grand Total** | **141758** | **142519** | **140209** | **96382** |  |
|  |  |  |  |  |  |

Supplement Table 2. (A) Breakdown of the Accident and Emergency attendees at Mater Dei Hospital between 2017 and April 2021 by specialities (B) Analysis of maximum likelihood parameter estimates for level shifts

(A)

| **2017** | **A&E** | **Dental** | **ENT** | **OBG** | **Ophth** | **Paeds** | **Psych** | **Grand Total** |
| --- | --- | --- | --- | --- | --- | --- | --- | --- |
| Jan | 7789 | 152 | 509 | 462 | 1063 | 1711 | 20 | **11706** |
| Feb | 6754 | 140 | 490 | 434 | 1102 | 1552 | 8 | **10480** |
| Mar | 7811 | 138 | 648 | 517 | 1291 | 1927 | 10 | **12342** |
| Apr | 7319 | 165 | 584 | 532 | 1243 | 1766 | 17 | **11626** |
| May | 7678 | 167 | 575 | 525 | 1288 | 1930 | 19 | **12182** |
| Jun | 7661 | 146 | 590 | 535 | 1319 | 1571 | 9 | **11831** |
| Jul | 8260 | 190 | 663 | 543 | 1402 | 1629 | 8 | **12695** |
| Aug | 8551 | 183 | 737 | 544 | 1253 | 1648 | 9 | **12925** |
| Sep | 7531 | 127 | 545 | 482 | 1203 | 1402 | 6 | **11296** |
| Oct | 7539 | 137 | 462 | 510 | 1204 | 1855 | 10 | **11717** |
| Nov | 7405 | 158 | 516 | 467 | 1167 | 1559 | 15 | **11287** |
| Dec | 7420 | 157 | 554 | 479 | 1114 | 1937 | 10 | **11671** |
| **Grand Total** | 91718 | 1860 | 6873 | 6030 | 14649 | 20487 | 141 | **141758** |
|  |  |  |  |  |  |  |  |  |
| **2018** | **A&E** | **Dental** | **ENT** | **OBG** | **Ophth** | **Paeds** | **Psych** | **Grand Total** |
| Jan | 8013 | 173 | 587 | 489 | 1200 | 1768 | 18 | **12248** |
| Feb | 7303 | 135 | 490 | 451 | 1056 | 1537 | 4 | **10976** |
| Mar | 8039 | 126 | 584 | 501 | 1324 | 1773 | 9 | **12356** |
| Apr | 7706 | 132 | 529 | 481 | 1228 | 1609 | 7 | **11692** |
| May | 7778 | 122 | 612 | 508 | 1255 | 1785 | 6 | **12066** |
| Jun | 7585 | 118 | 523 | 488 | 1136 | 1574 | 2 | **11426** |
| Jul | 8305 | 104 | 635 | 536 | 1266 | 1722 | 2 | **12570** |
| Aug | 8484 | 94 | 639 | 553 | 1240 | 1662 | 9 | **12681** |
| Sep | 7781 | 56 | 587 | 526 | 1166 | 1564 | 3 | **11683** |
| Oct | 7948 | 43 | 507 | 494 | 1161 | 2014 | 5 | **12172** |
| Nov | 7361 | 66 | 479 | 459 | 1124 | 1862 | 5 | **11356** |
| Dec | 7302 | 44 | 469 | 487 | 990 | 1997 | 4 | **74** |
| **Grand Total** | 93605 | 1213 | 6641 | 5973 | 14146 | 20867 | 74 | **142519** |
|  |  |  |  |  |  |  |  |  |
| **2019** | **A&E** | **Dental** | **ENT** | **OBG** | **Ophth** | **Paeds** | **Psych** | **Grand Total** |
| Jan | 7757 | 43 | 477 | 446 | 977 | 1887 | 4 | 11591 |
| Feb | 7192 | 60 | 464 | 441 | 993 | 1583 | 4 | 10737 |
| Mar | 7773 | 62 | 564 | 522 | 1267 | 1856 | 19 | 12063 |
| Apr | 7574 | 64 | 603 | 485 | 1122 | 1706 |  | 11554 |
| May | 7703 | 64 | 572 | 479 | 1157 | 1671 | 4 | 11650 |
| Jun | 7871 | 52 | 626 | 516 | 1145 | 1608 | 3 | 11821 |
| Jul | 8489 | 62 | 648 | 564 | 1234 | 1550 | 4 | 12551 |
| Aug | 8478 | 70 | 692 | 572 | 1199 | 1527 | 5 | 12543 |
| Sep | 7999 | 56 | 555 | 541 | 1077 | 1374 | 8 | 11610 |
| Oct | 7838 | 77 | 497 | 461 | 1161 | 1639 | 6 | 11679 |
| Nov | 7365 | 58 | 480 | 451 | 1045 | 1757 | 6 | 11162 |
| Dec | 7241 | 67 | 543 | 416 | 1014 | 1967 |  | 11248 |
| **Grand Total** | **93280** | **735** | **6721** | **5894** | **13391** | **20125** | **63** | **140209** |
|  |  |  |  |  |  |  |  |  |
| **2020** | **A&E** | **Dental** | **ENT** | **OBG** | **Ophth** | **Paeds** | **Psych** | **Grand Total** |
| Jan | 8126 | 79 | 578 | 453 | 1047 | 1975 | 6 | 12264 |
| Feb | 7207 | 47 | 491 | 413 | 1040 | 1653 | 2 | 10853 |
| Mar | 4790 | 27 | 249 | 306 | 554 | 680 | 3 | 6609 |
| Apr | 4202 | 64 | 137 | 333 | 439 | 310 | 4 | 5489 |
| May | 5159 | 88 | 209 | 344 | 551 | 464 | 8 | 6823 |
| Jun | 5936 | 122 | 280 | 368 | 740 | 564 | 3 | 8013 |
| Jul | 6623 | 118 | 352 | 429 | 847 | 800 | 3 | 9172 |
| Aug | 6060 | 59 | 361 | 340 | 675 | 688 | 1 | 8184 |
| Sep | 5673 | 60 | 296 | 365 | 645 | 568 | 13 | 7620 |
| Oct | 5434 | 59 | 249 | 289 | 623 | 652 | 7 | 7313 |
| Nov | 5171 | 68 | 202 | 235 | 589 | 632 | 8 | 6905 |
| Dec | 5371 | 80 | 232 | 273 | 594 | 584 | 3 | 7137 |
| **Grand Total** | **69752** | **871** | **3636** | **4148** | **8344** | **9570** | **61** | **96382** |
|  |  |  |  |  |  |  |  |  |
| **2021** | **A&E** | **Dental** | **ENT** | **OBG** | **Ophth** | **Paeds** | **Psych** | **Grand Total** |
| Jan | 5555 | 74 | 231 | 304 | 659 | 636 | 7 | 7466 |
| Feb | 5447 | 66 | 184 | 301 | 649 | 683 | 16 | 7346 |
| Mar | 5643 | 69 | 111 | 273 | 685 | 603 | 7 | 7391 |
| Apr | 6112 | 82 | 146 | 337 | 778 | 664 | 7 | 8126 |

(B)

| Category | Ratio | Lower Limit | Upper Limit | CHI sq. | p-value |
| --- | --- | --- | --- | --- | --- |
|  |  |  |  |  |  |
| A&E | 0.7 | 0.67 | 0.73 | 214.92 | <0.01 |
| Dental | 1.85 | 1.17 | 2.92 | 7 | <0.01 |
| ENT | 0.43 | 0.35 | 0.53 | 65.32 | <0.01 |
| OBG | 0.69 | 0.57 | 0.83 | 14.39 | <0.01 |
| Ophth | 0.61 | 0.53 | 0.69 | 55.99 | <0.01 |
| Paeds | 0.35 | 0.31 | 0.4 | 292.9 | <0.01 |
| Psych | 2.15 | 0.42 | 11.09 | 0.84 | 0.3588 |
| Grand Total | 0.63 | 0.61 | 0.66 | 505.64 | <0.01 |

Supplement Table 3. (A) Breakdown of the hospital admission to Mater Dei Hospital between 2017 and April 2021 by different categories (B) Analysis of maximum likelihood parameter estimates

(A)

|  | **2017** | **2018** | **2019** | **2020** | **2021** |
| --- | --- | --- | --- | --- | --- |
| **January** | **8472** | **8452** | **8659** | **8652** | **6310** |
| Day case | 2748 | 2617 | 2663 | 2715 | 1984 |
| Other | 305 | 443 | 426 | 549 | 347 |
| Elective | 1274 | 1333 | 1316 | 1290 | 792 |
| Emergency | 4145 | 4059 | 4254 | 4098 | 3187 |
| **February** | **7415** | **7784** | **7768** | **7900** | **6034** |
| Day case | 2578 | 2392 | 2490 | 2707 | 2055 |
| Other | 357 | 384 | 351 | 435 | 323 |
| Elective | 1015 | 1278 | 1271 | 1089 | 735 |
| Emergency | 3465 | 3730 | 3656 | 3669 | 2921 |
| **March** | **8341** | **8105** | **8221** | **5106** | **6016** |
| Day case | 2743 | 2520 | 2545 | 1366 | 1770 |
| Other | 378 | 397 | 411 | 356 | 352 |
| Elective | 1453 | 1329 | 1282 | 857 | 759 |
| Emergency | 3767 | 3859 | 3983 | 2527 | 3135 |
| **April** | **7526** | **7825** | **8008** | **3340** | **6360** |
| Day case | 2488 | 2681 | 2595 | 170 | 1993 |
| Other | 372 | 380 | 378 | 227 | 363 |
| Elective | 1284 | 1286 | 1320 | 522 | 1034 |
| Emergency | 3382 | 3478 | 3715 | 2421 | 2970 |
| **May** | **7918** | **8227** | **8405** | **4295** |  |
| Day case | 2653 | 2846 | 2799 | 665 |  |
| Other | 334 | 364 | 343 | 276 |  |
| Elective | 1377 | 1422 | 1405 | 653 |  |
| Emergency | 3554 | 3595 | 3858 | 2701 |  |
| **June** | **7271** | **7454** | **7616** | **5733** |  |
| Day case | 2335 | 2559 | 2554 | 1613 |  |
| Other | 289 | 353 | 419 | 300 |  |
| Elective | 1296 | 1177 | 1088 | 869 |  |
| Emergency | 3351 | 3365 | 3555 | 2951 |  |
| **July** | **7722** | **7926** | **8521** | **7088** |  |
| Day case | 2542 | 2636 | 2825 | 2417 |  |
| Other | 377 | 409 | 437 | 333 |  |
| Elective | 1333 | 1374 | 1483 | 1035 |  |
| Emergency | 3470 | 3507 | 3776 | 3303 |  |
| **August** | **7977** | **7811** | **8014** | **6533** |  |
| Day case | 2562 | 2425 | 2661 | 1935 |  |
| Other | 492 | 465 | 443 | 402 |  |
| Elective | 1288 | 1309 | 1202 | 1012 |  |
| Emergency | 3635 | 3612 | 3708 | 3184 |  |
| **September** | **7293** | **7346** | **7915** | **6255** |  |
| Day case | 2366 | 2313 | 2593 | 2005 |  |
| Other | 445 | 462 | 498 | 389 |  |
| Elective | 1174 | 1150 | 1260 | 846 |  |
| Emergency | 3308 | 3421 | 3564 | 3015 |  |
| **October** | **8105** | **8272** | **8597** | **6235** |  |
| Day case | 2737 | 2680 | 2990 | 1920 |  |
| Other | 441 | 465 | 474 | 351 |  |
| Elective | 1367 | 1410 | 1419 | 1096 |  |
| Emergency | 3560 | 3717 | 3714 | 2868 |  |
| **November** | **8305** | **8275** | **8321** | **5808** |  |
| Day case | 2814 | 2816 | 2672 | 1545 |  |
| Other | 514 | 437 | 500 | 317 |  |
| Elective | 1393 | 1454 | 1382 | 1126 |  |
| Emergency | 3584 | 3568 | 3767 | 2820 |  |
| **December** | **7313** | **7326** | **7573** | **5636** |  |
| Day case | 2107 | 2226 | 2223 | 1260 |  |
| Other | 404 | 369 | 417 | 326 |  |
| Elective | 1126 | 1068 | 1071 | 1104 |  |
| Emergency | 3676 | 3663 | 3862 | 2946 |  |
| **Grand Total** | **93658** | **94803** | **97618** | **72581** |  |
|  |  |  |  |  |  |

(B)

*Analysis Of Maximum Likelihood Parameter Estimates*

| *Parameter* |  | DF | Estimate | Standard Error | Wald 95% Confidence Limits | | Wald Chi-Square | Pr > ChiSq | | |
| --- | --- | --- | --- | --- | --- | --- | --- | --- | --- | --- |
| *Intercept adm* |  | 1 | 5.9863 | 0.0482 | 5.8918 | 6.0809 | 15413.8 |  | <.0001 |  |
|  | DAYCASE | 1 | 1.8345 | 0.0483 | 1.7397 | 1.9292 | 1440.83 |  | <.0001 |  |
| *adm* | Elective | 1 | 1.1340 | 0.0516 | 1.0328 | 1.2351 | 483.05 |  | <.0001 |  |
| *adm adm* | Emergency | 1 | 2.1844 | 0.0473 | 2.0916 | 2.2772 | 2129.54 |  | <.0001 |  |
|  | Other | 0 | 0.0000 | 0.0000 | 0.0000 | 0.0000 | . | . | | |
| *t* |  | 1 | 0.0242 | 0.0110 | 0.0026 | 0.0458 | 4.84 |  | 0.0277 |  |
| *ld*  *ld*adm* |  | 1 | -0.2690 | 0.0968 | -0.4586 | -0.0794 | 7.73 |  | 0.0054 |  |
|  | DAYCASE | 1 | -0.2516 | 0.1026 | -0.4527 | -0.0506 | 6.02 |  | 0.0142 |  |
| *ld*adm* | Elective | 1 | -0.1525 | 0.1095 | -0.3672 | 0.0622 | 1.94 |  | 0.1638 |  |
| *ld*adm ld*adm* | Emergency | 1 | -0.0115 | 0.0990 | -0.2056 | 0.1825 | 0.01 | 0.9074 | | |
|  | Other | 0 | 0.0000 | 0.0000 | 0.0000 | 0.0000 | . | . | | |
| *Scale* |  | 0 | 5.6262 | 0.0000 | 5.6262 5.6262 | |  |  | | |

ld: dummy variable for lockdown

Supplement Table 4. (A) Breakdown of the outpatient clinic attendees to Mater Dei Hospital between 2017 and April 2021 by different categories (B) Analysis of maximum likelihood parameter estimates

(A)

|  | **2017** | **2018** | **2019** | **2020** | **2021** |
| --- | --- | --- | --- | --- | --- |
| **January** | **41812** | **46008** | **43213** | **46104** | **34660** |
| Any | 6514 | 6341 | 4905 | 3681 | 2892 |
| Follow Up | 17076 | 19682 | 19344 | 21935 | 15984 |
| New Case | 7121 | 8107 | 7883 | 8234 | 6773 |
| Tel Cons | 0 | 0 | 0 | 0 | 1503 |
| Walk In | 11101 | 11878 | 11081 | 12254 | 7508 |
| **February** | **38291** | **41084** | **40816** | **42442** | **35064** |
| Any | 6269 | 5998 | 4829 | 3930 | 2463 |
| Follow Up | 15576 | 17078 | 17930 | 19258 | 15216 |
| New Case | 6731 | 7389 | 7531 | 7733 | 6653 |
| Tel Cons | 0 | 0 | 0 | 0 | 2354 |
| Walk In | 9715 | 10619 | 10526 | 11521 | 8378 |
| **March** | **44877** | **43332** | **42314** | **24469** | **36796** |
| Any | 7309 | 6549 | 5306 | 2180 | 2863 |
| Follow Up | 18096 | 17935 | 18517 | 10617 | 15333 |
| New Case | 7881 | 7481 | 7674 | 4544 | 6682 |
| Tel Cons | 0 | 0 | 0 | 0 | 3219 |
| Walk In | 11591 | 11367 | 10817 | 7128 | 8699 |
| **April** | **39324** | **44550** | **44132** | **10078** | **39371** |
| Any | 6355 | 6651 | 5250 | 343 | 2910 |
| Follow Up | 15912 | 18690 | 19312 | 4527 | 17086 |
| New Case | 6851 | 7733 | 7907 | 1291 | 7657 |
| Tel Cons | 0 | 0 | 0 | 0 | 2425 |
| Walk In | 10206 | 11476 | 11663 | 3917 | 9293 |
| **May** | **44691** | **46710** | **46244** | **16038** |  |
| Any | 7604 | 6569 | 5405 | 786 |  |
| Follow Up | 18197 | 19929 | 20539 | 7035 |  |
| New Case | 7511 | 8529 | 8483 | 2554 |  |
| Tel Cons | 0 | 0 | 0 | 0 |  |
| Walk In | 11379 | 11683 | 11817 | 5663 |  |
| **June** | **38230** | **39535** | **39310** | **26463** |  |
| Any | 5824 | 5089 | 3870 | 1534 |  |
| Follow Up | 16175 | 17046 | 17277 | 11682 |  |
| New Case | 6246 | 7428 | 7472 | 5207 |  |
| Tel Cons | 0 | 0 | 0 | 0 |  |
| Walk In | 9985 | 9972 | 10691 | 8040 |  |
| **July** | **41584** | **44759** | **47350** | **37189** |  |
| Any | 7170 | 6227 | 4198 | 2250 |  |
| Follow Up | 17363 | 18459 | 21656 | 17364 |  |
| New Case | 7016 | 8294 | 9162 | 7631 |  |
| Tel Cons | 0 | 0 | 0 | 123 |  |
| Walk In | 10035 | 11779 | 12334 | 9821 |  |
| **August** | **42244** | **43460** | **43007** | **28256** |  |
| Any | 5912 | 4835 | 3002 | 1806 |  |
| Follow Up | 18088 | 18737 | 19772 | 12612 |  |
| New Case | 7966 | 8193 | 8667 | 5331 |  |
| Tel Cons | 0 | 0 | 0 | 816 |  |
| Walk In | 10278 | 11695 | 11566 | 7691 |  |
| **September** | **39770** | **38875** | **43248** | **34686** |  |
| Any | 6176 | 4541 | 3536 | 2243 |  |
| Follow Up | 16271 | 16932 | 20128 | 16269 |  |
| New Case | 7273 | 7217 | 8294 | 6669 |  |
| Tel Cons | 0 | 0 | 0 | 942 |  |
| Walk In | 10050 | 10185 | 11290 | 8563 |  |
| **October** | **45464** | **47038** | **48709** | **37210** |  |
| Any | 7403 | 5672 | 4350 | 2845 |  |
| Follow Up | 18674 | 20526 | 22467 | 17221 |  |
| New Case | 8116 | 8888 | 9480 | 7362 |  |
| Tel Cons | 0 | 0 | 0 | 1374 |  |
| Walk In | 11271 | 11952 | 12412 | 8408 |  |
| **November** | **47072** | **45322** | **45089** | **33651** |  |
| Any | 7233 | 5794 | 4161 | 2844 |  |
| Follow Up | 19628 | 19117 | 20838 | 15334 |  |
| New Case | 8548 | 8507 | 8812 | 6805 |  |
| Tel Cons | 0 | 0 | 0 | 1590 |  |
| Walk In | 11663 | 11904 | 11278 | 7078 |  |
| **December** | **32457** | **31862** | **35157** | **28175** |  |
| Any | 4876 | 3935 | 3062 | 2053 |  |
| Follow Up | 13315 | 13699 | 16304 | 12882 |  |
| Tel Cons | 0 | 0 | 0 | 5455 |  |
| New Case | 5848 | 5927 | 6760 | 1463 |  |
| Walk In | 8418 | 8301 | 9031 | 6322 |  |
| **Grand Total** | **495816** | **512535** | **518589** | **364761** |  |

(B)

**Analysis Of Maximum Likelihood Parameter Estimates**

| *Parameter* |  | DF | Estimate | Standard Error | Wald 95% Confidence Limits | | Wald Chi-Square | Pr > ChiSq | | |
| --- | --- | --- | --- | --- | --- | --- | --- | --- | --- | --- |
| *Intercept*  *t* |  | 1 | 7.6373 | 0.4156 | 6.8228 | 8.4518 | 337.73 | <.0001 | | |
|  |  | 1 | 0.3003 | 0.1037 | 0.0971 | 0.5035 | 8.39 |  | 0.0038 |  |
| *opa* | Any | 1 | 1.2815 | 0.4176 | 0.4631 | 2.0999 | 9.42 |  | 0.0021 |  |
| *opa opa* | Follow Up | 1 | 2.0802 | 0.4163 | 1.2643 | 2.8962 | 24.97 |  | <.0001 |  |
|  | New Case | 1 | 1.2384 | 0.4173 | 0.4206 | 2.0563 | 8.81 |  | 0.0030 |  |
| *opa* | Tel Cons | 1 | -6.2220 | 1.6595 | -9.4746 | -2.9694 | 14.06 |  | 0.0002 |  |
| *t*opa t*opa* | Any | 1 | -0.5139 | 0.1065 | -0.7226 | -0.3052 | 23.29 |  | <.0001 |  |
|  | Follow Up | 1 | -0.2381 | 0.1045 | -0.4429 | -0.0333 | 5.19 |  | 0.0227 |  |
| *t*opa* | New Case | 1 | -0.2498 | 0.1056 | -0.4568 | -0.0428 | 5.60 |  | 0.0180 |  |
| *t*opa ld* | Tel Cons | 1 | 1.2748 | 0.4112 | 0.4689 | 2.0807 | 9.61 |  | 0.0019 |  |
|  |  | 1 | -2.6976 | 0.3194 | -3.3236 | -2.0716 | 71.34 |  | <.0001 |  |
| *t*ld* |  | 1 | 0.5992 | 0.0840 | 0.4345 | 0.7639 | 50.84 |  | <.0001 |  |
| *ld*opa* | Any | 1 | -1.6081 | 0.7479 | -3.0740 | -0.1422 | 4.62 |  | 0.0315 |  |
| *ld*opa* | Follow Up | 1 | 0.3088 | 0.4153 | -0.5053 | 1.1228 | 0.55 | 0.4572 | | |
| *ld*opa ld*opa* | New Case | 1 | -0.0041 | 0.5189 | -1.0211 | 1.0129 | 0.00 | 0.9937 | | |
|  | Tel Cons | 0 | 0.0000 | 0.0000 | 0.0000 | 0.0000 | . | . | | |
| *t*ld*opa* | Any | 1 | 0.4199 | 0.1958 | 0.0362 | 0.8037 | 4.60 |  | 0.0320 |  |
| *t*ld*opa t*ld*opa* | Follow Up | 1 | -0.0844 | 0.1095 | -0.2991 | 0.1303 | 0.59 | 0.4412 | | |
|  | New Case | 1 | 0.0064 | 0.1368 | -0.2618 | 0.2745 | 0.00 | 0.9630 | | |
| *t*ld*opa* | Tel Cons | 0 | 0.0000 | 0.0000 | 0.0000 | 0.0000 | . | . | | |
| *Scale* |  | 0 | 14.1064 | 0.0000 | 14.1064 14.1064 | |  |  | | |

Supplement Figure 1. (A) A&E attendance by vaccination dynamics and (B) Analysis of maximum likelihood parameter estimates

| **Analysis Of Maximum Likelihood Parameter Estimates** | | | | | | | |
| --- | --- | --- | --- | --- | --- | --- | --- |
| **Parameter** | **DF** | **Estimate** | **Standard** | **Wald 95% Confidence Limits** | | **Wald Chi-Square** | **Pr > ChiSq** |
|  |  |  | **Error** |  |  |  |  |
| **Intercept** | 1 | 8.5973 | 0.0178 | 8.5624 | 8.6322 | 233112 | <.0001 |
| **vac** | 1 | 0.6048 | 0.166 | 0.2795 | 0.9301 | 13.28 | 0.0003 |
| **Scale** | 0 | 1.7153 | 0 | 1.7153 | 1.7153 |  |  |

Supplement Figure 2. (A) A&E attendance by vaccination dynamics with a two week lag and (B) Analysis of maximum likelihood parameter estimates

(A)

(B)

| **Analysis Of Maximum Likelihood Parameter Estimates** | | | | | | | |
| --- | --- | --- | --- | --- | --- | --- | --- |
| **Parameter** | **DF** | **Estimate** | **Standard** | **Wald 95% Confidence Limits** | | **Wald Chi-Square** | **Pr > ChiSq** |
|  |  |  | **Error** |  |  |  |  |
| **Intercept** | 1 | 8.6034 | 0.0152 | 8.5737 | 8.6331 | 321716 | <.0001 |
| **vacl1m** | 1 | 0.7976 | 0.002 | 0.0041 | 0.0119 | 15.97 | <.0001 |
| **Scale** | 0 | 1.5815 | 0 | 1.5815 | 1.5815 |  |  |
